# Forgetting phenomena in the Iowa Gambling Task: a new computational model among diverse participants

**DOI:** 10.3389/fpsyg.2025.1510151

**Published:** 2025-06-05

**Authors:** Tiancheng Yang, Chenghan Xie, Xuehe Wang

**Affiliations:** ^1^School of Artificial Intelligence, Sun Yat-sen University, Zhuhai, China; ^2^College of Engineering, Department of Information Engineering, The Chinese University of Hong Kong, Hong Kong, China

**Keywords:** Forgetting Phenomena, Iowa Gambling Task, Exploitation and Exploration with Forgetting model, Sequential Exploration Decay, Forgetting Interval

## Abstract

**Introduction:**

The Iowa Gambling Task (IGT) is a widely used paradigm for evaluating decision-making and executive functioning, yet existing computational models seldom account for the phenomenon of forgetting, which is critical to understanding dynamic decision processes.

**Methods:**

We developed the Exploitation and Exploration with Forgetting (EEF) model, which integrates a dynamic forgetting parameter (λ) and participants' first-choice priors into a unified reinforcement-learning framework. The EEF model was fitted to choice data from 504 healthy individuals performing the standard 100-trial IGT. Model performance was assessed via goodness-of-fit comparisons (BIC/AIC/Free Energy), parameter- and model-recovery simulations, and behavioral validation.

**Results:**

Across multiple cohorts, the EEF model achieved superior fit relative to five established models. We introduce two novel metrics—Sequential Exploration Decay (SED) and Forgetting Interval (FI)—to quantify how forgetting shapes exploratory behavior. The EEF model's SED and FI values closely matched empirical data, and further analyses revealed systematic effects of age and gambling frequency on forgetting and decision strategies.

**Discussion:**

Our findings underscore the fundamental role of forgetting in complex decision-making environments. By explicitly modeling information decay, the EEF framework offers novel insights into cognitive dynamics across the lifespan and behavioral contexts, and provides a parsimonious yet powerful tool for future computational and empirical research.

## 1 Introduction

Forgetting is a crucial concept in psychology, as it affects both memory capacity and key cognitive activities such as learning and decision-making. In the field of psychology, researchers have begun to recognize that forgetting is not merely a manifestation of memory impairment but rather an essential capability for proper brain function (Nørby, 2015). Studies have shown that forgetting facilitates the optimization of information storage and utilization. When forgetting occurs, the brain releases more cognitive resources for information processing, enabling individuals to free themselves from overwhelming information (Gaissmaier et al., 2008). Professor Blake Richards, who specializes in the study of neural circuits, once remarked: “Our ability to generalize new experiences is, at least in part, due to the fact that our brains engage in controlled forgetting.” He posited that forgetting serves as a mechanism for the brain to prevent overfitting (Gravitz, 2019).

Although significant progress has been made in recent years in understanding the mechanisms and functions of forgetting, research integrating forgetting into cognitive decision-making models remains relatively limited. In the field of cognitive science, researchers typically employ a series of standardized cognitive paradigms to investigate information processing, risk assessment, and reward mechanisms during decision-making processes. These paradigms help us understand how the brain makes decisions in complex situations (Serrano and Alacreu-Crespo, 2022). The Iowa Gambling Task (IGT) is one of the most widely used experimental paradigms in this context. First introduced by Bechara et al. (1994), the IGT was designed to evaluate an individual's decision-making abilities when faced with risk and uncertainty. It is now extensively applied in cognitive psychology and neuropsychology and is considered an essential tool for measuring cognitive decision-making. By simulating real-life decision-making scenarios, the IGT assesses individuals' decision making under uncertainty; it has been widely interpreted as tapping emotion-related decision processes (Bechara et al., 1994). Furthermore, the IGT has been widely used in clinical settings to assess decision-making deficits in patients with various psychological and neurological disorders, especially in populations with brain injuries, substance abuse, neurological diseases, and mental disorders (Yechiam et al., 2005; Hu et al., 2023).

In the IGT, participants are required to make selections from four card decks (A, B, C, and D), each of which has different reward and penalty structures. Each choice may result in either a reward or a loss. The goal of the task is to maximize the total net gain within a limited number of trials. Among these decks, A and B offer higher immediate rewards but lead to net losses in the long run due to their larger long-term penalties, potentially resulting in negative overall gains. In contrast, C and D provide lower immediate rewards but have smaller penalties, leading to positive gains in the long run (Hu et al., 2023). In the original study by Bechara et al. (1994), the experiment was designed with a total of 100 choices. The payoff scheme for the four decks (A, B, C, and D) is shown in Table 1.

**Table 1:** Payoff scheme used in the Iowa Gambling Task by Bechara et al. (1994).

	**A**	**B**	**C**	**D**
Profit per choice	100	100	50	50
Number of losses per 10 choices	5	1	5	1
Possible loss amounts	–150	–1,250	–25	–250
	–200		–50	
	–250		–75	
	–300			
	–350			
Total return after 10 choices	–250	–250	250	250

When selecting Deck A continuously for 10 trials, there will be 5 occurrences of random losses ranging from $150 to $350. Similarly, for every 10 consecutive selections of Deck B, there will be 1 occurrence of a large loss of $1,250. In contrast, selecting Deck C or D yields a gain of $50 each time. For every 10 consecutive selections of Deck C, there will be 5 occurrences of a total fixed loss of $250; likewise, for every 10 consecutive selections of Deck D, there will be 1 fixed loss of $250. Based on the expected value calculated over 10 choices, choosing Deck A or B results in an average loss of $250, whereas choosing Deck C or D is expected to yield an average positive gain of $250 (Bechara et al., 1994).

As summarized in Table 1, Decks A and B yield a nominal profit of $100 on every draw but conceal larger long-term losses, whereas Decks C and D pay only $50 per draw yet accrue a positive net return over a ten-draw horizon. This trade-off between high-reward/high-risk and low-reward/low-risk options creates the classic exploitation–exploration dilemma at the heart of the IGT.

This study laid an important foundation for understanding how humans make decisions when confronted with uncertainty and risk. Although subsequent IGT experiments and their variants may have adjusted the total number of trials and reward mechanisms, the fundamental essence of the task remains unchanged. This consistency ensures the comparability of research findings and allows for in-depth analysis of decision-making behaviors across different experimental conditions.

Traditional modeling approaches for analyzing IGT data primarily focus on value-based learning and decision-making. Common models include the Expected Value (EV) model, the Prospect Valence Learning (PVL) model, and a hybrid version combining EV and PVL—the PVL-Delta model. Although these models have made progress in quantifying the weights of gains and losses during decision-making processes, they often overlook other critical dynamics in the decision-making process (Ahn et al., 2008; Busemeyer and Stout, 2002). As a result, their explanatory power and application scope are somewhat limited. With the advancement of research, more modeling approaches that emphasize exploratory behavior have been proposed. Among these, Directed Exploration (DE) offers a new perspective on IGT research by proposing an exploration strategy that integrates information preferences into decision-making models. This strategy explicitly prioritizes information gathering rather than merely maximizing gains (Wilson et al., 2014). Building on this foundation, the Value and Sequential Exploration (VSE) model, designed by Ligneul (2019), introduces sequential exploration dynamics to capture exploitation, random exploration, and sequential exploration in the IGT while maintaining sensitivity to existing rewards.

To further enhance the explanatory power and applicability of models, some researchers have begun incorporating additional elements into IGT decision-making models. The Value Plus Perseveration (VPP) model is one such attempt, which not only considers the weights of gains but also introduces the concept of perseveration in decision-making (Worthy et al., 2013). However, the VPP model requires eight parameters, which is relatively complex given the number of trials, making it challenging in terms of both application and interpretation, especially regarding the cognitive validity of these parameters (Konstantinidis et al., 2014). On the other hand, the Outcome Representation Learning (ORL) model also attempts to capture more decision-making behaviors by increasing model complexity. Nevertheless, its perseveration module does not integrate reward magnitude and reward probability into the estimation of reward expectations, which is considered inconsistent with fundamental theories in behavioral economics (Haines et al., 2018). Although the aforementioned models offer new theoretical pathways for interpreting IGT, current IGT models rarely consider forgetting as a core variable, particularly in the context of complex, repeated decision-making environments.

By effectively quantifying forgetting, we can reveal how individuals adjust their cognitive strategies when confronted with constantly changing environmental information, which has significant theoretical and practical implications for research ranging from cognitive health to neuropsychiatric disorders. To meet this demand, we propose a new computational model called Exploitation and Exploration with Forgetting (EEF), which, for the first time, introduces a parameter specifically designed to quantify forgetting ability within the context of the IGT.

### 1.1 Main contributions

We present a four-parameter Exploitation–Exploration with Forgetting (EEF) model that integrates an *individualized forgetting rate* into the reinforcement-learning update, thereby capturing person specific information loss while remaining highly parsimonious. Deck-specific initial weights are derived directly from the empirical first-choice frequencies, anchoring the model in observed behavior rather than arbitrary priors. To probe information decay dynamics we introduce two complementary indices—Sequential Exploration Decay (SED) and Forgetting Interval (FI)—which quantify, respectively, the rate at which exploratory value wanes and the typical lag between informative choices. In addition, the EEF model was fitted to data from healthy young adults as well as two heterogeneous groups—individuals with gambling disorder and older adults—and achieved the best (or statistically comparable) BIC and AIC scores across these cohorts while remaining competitive on the free energy criterion. Parameter-recovery simulations yielded high correlations between generating and recovered values, confirming that the model's parameters are both identifiable and psychologically interpretable.

This study provides a more precise and in-depth methodology for modeling and understanding complex decision-making behaviors. Particularly in terms of cognitive validity, our research shows that the forgetting parameter can effectively capture individuals' information processing characteristics during decision-making and predict future choice behavior. Furthermore, we have enhanced the previously established open-source toolbox, making this model widely applicable to various research contexts and enabling researchers to more efficiently analyze and understand the phenomenon of forgetting in the IGT (Ligneul, 2019).

## 2 Descriptions of Exploitation and Exploration with Forgetting model

In the IGT, each individual's choice patterns across different card decks reflect their risk preferences, outcome evaluations, and how they learn and adjust their strategies based on experience. Researchers use computational cognitive models to simulate human decision-making processes when faced with uncertainty and potential risks, helping to elucidate the cognitive mechanisms underlying decision behaviors. In studying decision-making behaviors, normative decision theory adopts a top-down approach, using mathematical analyses to provide guidance for optimal decision-making. This theory is based on expected utility theory, emphasizing the maximization of expected utility when making rational decisions under conditions of uncertainty and risk (Morelli et al., 2022). However, real-world decisions often deviate from these rational standards, as decisions are typically made under conditions of insufficient information or uncertainty. To improve model performance, it is essential to consider the limitations of cognitive systems (e.g., limited attention and memory resources) and account for certain biases and irrational factors when constructing computational models.

Our Exploitation and Exploration with Forgetting (EEF) model builds upon the concepts of previous models and primarily consists of two components: an exploitation module and an exploration module. The exploitation module updates by analyzing recent gain and loss data associated with each card deck, while the exploration module adjusts by monitoring the recent selection frequencies of each option to capture behavioral patterns corresponding to sequential exploration dynamics observed in the IGT. The dynamic management of memory and forgetting is a fundamental element of human decision-making processes. Distinguishing itself from other models, the EEF model introduces an innovative “individualized forgetting parameter”. This mechanism not only influences the retention of information during exploitation but also regulates information updating in exploratory behavior. Specifically, in our EEF model, the forgetting parameter λ plays a crucial role in updating the weights of both exploitation and exploration. It controls the rate of information updating, reflecting the extent to which a person's decision-making is influenced only by recent rounds or information, rather than by all accumulated experiences. By adjusting the λ parameter, the model is able to capture individual differences in behavioral patterns when faced with decision-making scenarios.

Importantly, unlike the fixed geometric decay in PVL-Δ and VSE, our single forgetting parameter λ both modulates exploitation and exploration, updates interactively on each trial, and can be read as an individual's short-term memory horizon—thereby avoiding potential misunderstandings about scope, dynamics, or interpretability.

In addition, our model innovatively incorporates the initial preference knowledge of IGT participants, based on their first-choice patterns, to enhance the consistency between the model and human decision-making patterns. In this section, we will provide a detailed explanation of the EEF model, including the specific equations and parameters involved.

### 2.1 Exploitation with forgetting in IGT

The Exploitation and Exploration with Forgetting (EEF) model consists of two main components: an exploitation module and an exploration module. The exploitation module is conceptually similar to the core ideas in the PVL and VSE models, both of which primarily base their computations on gain and loss utilities. However, our model does not include the “loss aversion” parameter from the PVL model or the decay parameter from the VSE model. The EEF model specifically introduces two key control variables:

- **Sensitivity Control Variable** **θ** **(ranging between** **0** **and** **1):** Adjusts the impact of gains and losses on utility calculations.
- **Forgetting Parameter** **λ** **(ranging between** **0** **and** **1):** Represents the degree of information forgetting.

In each trial, the exploitation weight of each deck *d*, *d*∈{*A, B, C, D*}, is updated according to the following equation:


$$\begin{array}{l} {\text{Exploitation}}_{d}\left(t+1\right)=\left(1-\lambda \right){\text{Exploitation}}_{d}\left(t\right) \end{array}$$ (1)

Exploitation_*d*_(*t*) represents the exploitation weight of deck *d* at time *t*. Equation 1 describes how the exploitation weight of deck *d* is updated when it is not selected. Here, the weight gradually decreases at a rate controlled by the forgetting parameter λ, simulating the effect of forgetting—that is, the gradual decay of unused information.


$$\begin{array}{lll} V\left(t\right) & = & \text{Gain}{\left(t\right)}^{\theta }-\text{Loss}{\left(t\right)}^{\theta } \end{array}$$ (2)

$$\begin{array}{lll} {\text{Exploitation}}_{d}\left(t+1\right) & = & \left(1-\lambda \right){\text{Exploitation}}_{d}\left(t\right)+V\left(t\right) \end{array}$$ (3)

Equation 2 calculates the value *V*(*t*) at time *t*, where θ modulates the sensitivity to gains and losses. Equation 3 illustrates how, when deck *d* is selected, the exploitation weight is updated based on the recent monetary feedback. In the exploitation module, an increase in the forgetting parameter λ indicates faster forgetting, meaning that previous information is lost more rapidly. In other words, as an individual's level of forgetting increases, the emphasis in exploitation weight updates shifts from the prior exploitation weight to the current value incentive.

### 2.2 Exploration with forgetting in IGT

Exploration weight represents the gradually increasing attractiveness of a specific deck when it has not been selected for a period of time. The exploration weight is an independent function separate from the exploitation weight, meaning it is not influenced by the monetary feedback experienced during the IGT. Previous models have used the delta rule combined with a learning rate and exploration reward parameter to simulate sequential exploration behaviors by adjusting the weights of unselected decks (Ligneul, 2019). In our study, we design the increase in exploration weight to be closely related to the forgetting process, with a particular focus on the impact of forgetting on exploratory behaviors.

The exploration weight is governed by the following equations:


$$\begin{array}{lll} {\text{Exploration}}_{d}\left(t+1\right) & = & 0 \end{array}$$ (4)

$$\begin{array}{lll} {\text{Exploration}}_{d}\left(t+1\right) & = & \lambda {\text{Exploration}}_{d}\left(t\right)+\left(1-\lambda \right)\phi \end{array}$$ (5)

Exploration_*d*_(*t*) represents the exploration weight of deck *d* at time *t*. Equation 4 is the exploration weight update equation for a selected deck, indicating that the exploration weight of a selected deck is reset to zero. In contrast, Equation 5 includes the individual forgetting parameter λ and the exploration incentive parameter ϕ (ranging from −5 to 5). This equation describes that, starting from an initialized exploration weight, consistent with the cognitive reinforcement mechanisms observed in Yechiam et al. (2010), if a deck remains unselected over time, its exploration weight will gradually approach ϕ under the influence of exploration incentives, encouraging (if ϕ is positive) or discouraging (if ϕ is negative) the agent to re-explore options that have been ignored for a long period.

The equation also suggests that in groups with a high level of forgetting, the effect of exploration incentives may be weaker. In high-forgetting groups, individuals are inclined to ignore or quickly forget past information, making them more likely to forget the information related to “how long an option has been unchosen” when making decisions, causing the update of exploration weight to depend more on previous weights rather than on exploration incentives. To consider an extreme case, when the forgetting rate is 1, it implies that an individual with only memory of the current trial completely disregards past information and makes choices based solely on the rewards received in the current trial. In this scenario, the exploration weight remains unchanged from the initial value of zero, and each choice relies exclusively on the utility calculation of the exploitation weight. This distinction illustrates the different ways in which λ impacts exploration and exploitation weights.

### 2.3 Consistency parameter and decision probability computation

After calculating the exploitation weight and exploration weight for each deck using the exploitation and exploration modules, the EEF model determines the probability of selecting each deck using the following equation:


$$\begin{array}{l} P\left(\text{Choice}=d\right)=\frac{e^{\left({\text{Exploration}}_{d}+{\text{Exploitation}}_{d}\right)\times \text{Consistency}}}{\sum_{i=1}^{4}e^{\left({\text{Exploration}}_{i}+{\text{Exploitation}}_{i}\right)\times \text{Consistency}}} \end{array}$$ (6)

The EEF model follows a similar approach to the PVL model in generating choice probabilities (Steingroever et al., 2013). This equation treats the decision-making process as a stochastic process regulated by a consistency parameter (subsequently referred to as “*C*”), which adjusts the randomness of choice behavior. In statistical physics, *C* is analogous to the “inverse temperature” parameter β, which controls the probability distribution of state selection. In decision-making models, an increase in *C* indicates that the choice process is more influenced by the combined effect of the computed exploration and exploitation weights, thereby reducing randomness and increasing reliance on these weights.

Notably, the value of *C* is derived from a transformation of the inverse temperature parameter β, with the specific transformation formula defined as *C* = 3^β^ − 1, where β ranges from 0 to 5. The model ultimately converts these weights into choice probabilities using the softmax function, ensuring that the probability distribution is normalized (i.e., the sum of the probabilities for all options equals 1). This method effectively balances the impact of randomness and the influence of weights, ensuring that the model's choice behavior not only reflects the computed weights but also adheres to the statistical characteristics of a stochastic process.

### 2.4 IGT first choice: foundation of model prior knowledge

The structure of the IGT allows researchers to observe and analyze participants' decision-making patterns across multiple choices, which gradually become apparent as the task progresses. At the beginning of this sequence of decisions, the first choice plays a unique role. The first choice is made by participants without a full understanding of the long-term consequences of each deck, revealing their initial risk preferences and intuitive judgments. After constructing the core framework of the EEF model, we noted that, despite extensive research on the IGT, few studies have thoroughly explored participants' first-choice behaviors within the task, and it is even rarer to see these behaviors used as the foundation for model construction. Thus, this study aims to fill this gap by conducting an in-depth analysis of first choices in the IGT, with the goal of improving the model's parameter recovery when simulating human decision-making behavior.

Specifically, we seek to investigate first-choice patterns through a comprehensive dataset to provide prior knowledge that enhances model performance. We utilized a dataset containing 504 healthy participants (Steingroever et al., 2015), drawn from ten independent studies using standard or variant IGT payoff schemes, encompassing the classic 100-trial version of the task.

**Figure 1:**
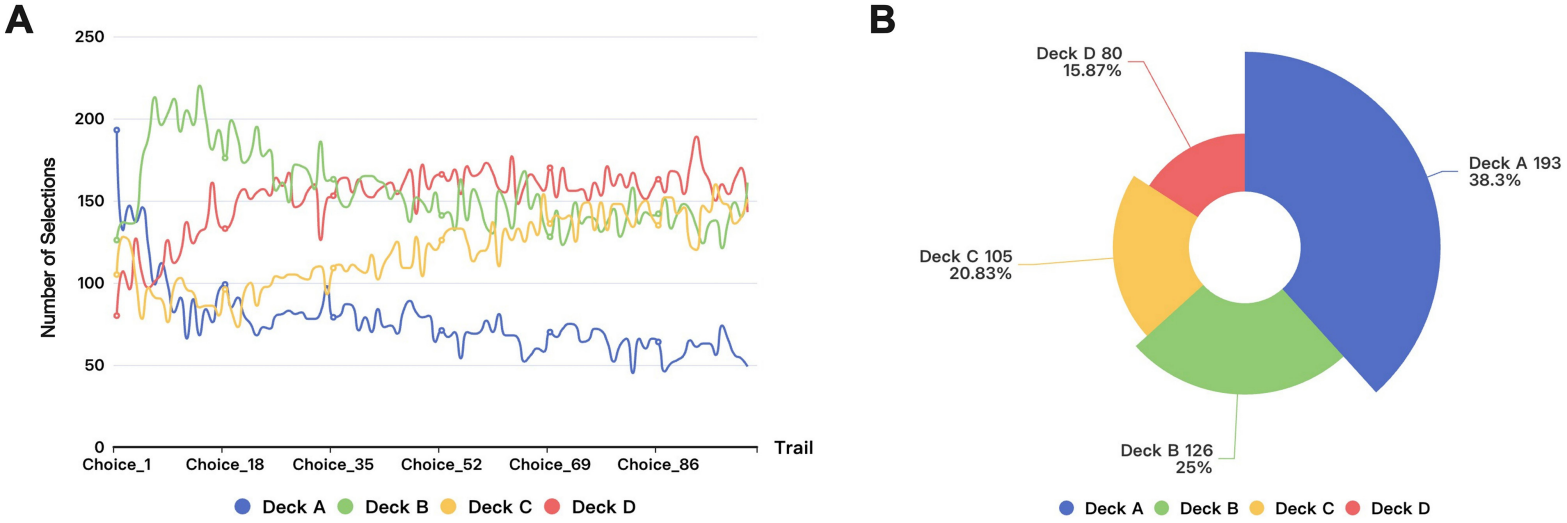
Visualization of the selection of 504 participants in IGT: **(A)** Round selection in IGT. **(B)** The first round section in IGT.

Figure 1 presents our visualization analysis of the choices made by 504 participants across each trial in the dataset (Steingroever et al., 2015). Figure 1A shows the choice frequency for each option across 100 trials by 504 participants, while Figure 1B reveals distinct preference patterns for card decks A, B, C, and D in the first round of the IGT. Statistical analysis indicates that Deck A is the most popular option, with a selection probability of 38.29%, chosen 193 times. This is followed by Deck B, which was chosen 126 times, accounting for 25.00% of the total choices. In contrast, Decks C and D have lower selection probabilities, at 20.83% (105 choices) and 15.87% (80 choices), respectively. The chi-square test further reinforces the significance of this pattern. The resulting chi-square statistic reached 68.46, with a corresponding *p*-value of 9.13 × 10^−15^. This *p*-value is far below the 0.05 threshold, clearly indicating that the observed choice frequencies differ significantly from the theoretical random probability of each option (25%). This suggests that under conditions of incomplete information, participants display a pronounced initial preference for certain options, particularly Deck A. Such a choice bias may be related to participants' intuitive judgments and their varying perceptions of risk, leading to the formation of preferences for certain decks in the early stages of decision-making.

The model's prior knowledge reflects the initial beliefs or experiences that humans rely on when making decisions. Incorporating this knowledge into the model allows it to better simulate human intuition and preferences, thereby enhancing its predictive accuracy and alignment with human decision-making patterns. To improve the model's fit to human decision-making behaviors, we incorporated the observed frequencies of participants' initial choices as prior knowledge in the model.

As previously mentioned, the EEF model combines the derived exploitation and exploration weights with a consistency parameter *C* and transforms them into choice probabilities using the softmax function. Thus, we can reverse-transform the observed choice frequencies from earlier through the softmax function (setting β = 3) to compute the initial weight for each deck, which serves as the model's prior knowledge.

The initial weights of the four card decks are calculated as follows:


$$\begin{array}{l} w_{\text{ini}}=\left[0.0184,0.0020,-0.0050,-0.0155\right] \end{array}$$ (7)

This initial weight serves as the model's starting condition and is combined with the weights subsequently derived from the exploitation and exploration strategies to simulate participants' decision-making behaviors in the Iowa Gambling Task. By doing so, the model is provided with a more reasonable starting condition, allowing it to capture participants' intuitive tendencies during the early stages of decision-making.

More than a mere *post-hoc* adjustment, embedding the empirically observed first-choice bias as a prior state is not a generic *post-hoc* tuning device but an organic element of the EEF's forgetting architecture. The vector ***w***_ini_ constitutes an initial memory trace that decays trial by trial at the individual specific forgetting rate λ, entering the same update equations as all subsequent experience. Benchmark models that lack an explicit memory-decay or exploration channel (e.g., EV, PVL-Δ) cannot accommodate such transient priors without redefining their core equations, which would blur the theoretical distinctions under comparison. For this reason the first-choice prior is implemented only in the EEF model, where it naturally complements the joint principles of forgetting and dynamic memory renewal.

## 3 Materials and methods

### 3.1 Data sources

To systematically analyze IGT decision-making patterns and validate the empirical fit of the new computational model, we selected several key datasets, all of which used the IGT as a research tool. The following section details these datasets and their applications in this study.

#### 3.1.1 Iowa Gambling Task performance dataset

This study primarily utilizes the dataset containing 504 healthy participants mentioned earlier as the basis for validating the EEF model. This dataset is integrated from 10 independent studies, with an original sample size of 617 healthy individuals, defined as participants without reported neurological disorders. The data were collected from studies using either the traditional IGT payoff scheme or its variants, with all data obtained through computerized versions of the IGT, covering different samples ranging from 95 to 150 trials. Specifically, we selected data from studies using the standard 100-trial version, encompassing a total of 504 participants. This dataset provides detailed records of each participant's choices in every trial and their corresponding reward and loss outcomes (Steingroever et al., 2015). Detailed information on the studies included in the dataset is presented in Table 2.

**Table 2:** Iowa Gambling Task performance dataset (Steingroever et al., 2015).

**Study**	**Number of participants**	**Number of trials**	**Demographics**
Horstmann et al. (2012)	162	100	*M* = 25.6 years (*SD* = 4.9), 82 female
Kjome et al. (2010)	19	100	*M* = 33.9 years (*SD* = 11.2), 6 female
Maia and McClelland (2004)	40	100	Undergraduate students
Premkumar et al. (2008)	25	100	*M* = 35.4 years (*SD* = 11.9), 9 female
Steingroever et al. (2013)	70	100	*M* = 24.9 years (*SD* = 5.8), 49 female
Wood et al. (2005)	153	100	*M* = 45.25 years (*SD* = 27.21), wide age range
Worthy et al. (2013)	35	100	Undergraduate students, 22 female

The original article provided detailed participant information, including age, occupation, and gender distribution. These data were collected by Annette Horstmann and were first published in the study by Steingroever et al. (2013). A subset of this dataset is also included in the study by Horstmann et al. (2012). Participants in the dataset are divided into two main age groups. The first 90 participants are young adults, aged between 18 and 40 years, with an average age of 23.04 years (*SD* = 5.88). The next 63 participants are older adults, aged between 61 and 88 years, with an average age of 76.98 years (*SD* = 5.20).

The seven contributing studies span laboratory, online, and clinical contexts and cover a broad age range (18–88 years). This heterogeneity reduces sampling bias and enables us to test whether each competing model generalizes across demographic variability.

#### 3.1.2 IGT in gambling behaviors dataset

We also included a dataset focused on decision-making behaviors among individuals with varying gambling frequencies, originally consisting of 147 participants (Kildahl et al., 2020). Due to missing critical condition labels and gambling frequency information, we excluded some participants and ultimately used data from 139 participants for analysis.

Participants were recruited through the participant system of Aarhus University's Cognition and Behavior Laboratory, with the majority being students (accounting for 84%). To meet the participation criteria, all participants must have engaged in at least one gambling activity within the past three months and be at least 18 years old. Participants in the dataset were divided into four groups based on their self-reported gambling frequency: less than once per month (38 participants), at least once per month (45 participants), once per week (29 participants), and multiple times per week (27 participants). The experiment was conducted through the Gorilla online experimental platform, requiring participants to complete the task in a quiet environment in a single session. The ages of the participants primarily ranged from 27 years and below, with the majority being between 18 and 22 years old (71 participants), followed by 23 to 27 years old (56 participants), and a small number of participants over 28 years old.

### 3.2 Model evaluation and comparison methods

As experimental data and theoretical understanding continue to advance, the validation and comparison of decision-making simulation methods have become increasingly important. This section introduces and compares several decision-making simulation methods used for the IGT. In particular, we introduce two new evaluation metrics—Sequential Exploration Decay (SED) and Forgetting Interval (FI)—which help us further investigate the effectiveness of various methods in capturing forgetting and exploratory behaviors.

#### 3.2.1 Sequential exploration decay

In a previous study, Ligneul (2019) proposed an index for quantifying sequential exploration, referred to as the “SE index.” This index measures the occurrence of directed exploration by calculating the probability that participants select four different decks consecutively over four trials. Their study found that the actual frequency of SE events was 11.1%, significantly higher than the expected random exploration frequency of 9.38% (binomial test: *p* < 10^−10^), indicating that participants tend to engage in more exploratory choices. Although the SE index effectively captures directed exploration behaviors in the IGT, it primarily reflects overall exploration patterns and cannot finely distinguish between behavioral changes in the early and late stages of the task, nor can it capture changes in information value decay across different phases.

In the IGT, even though participants may have already identified the long-term gains or risks associated with certain decks, forgetting and other psychological biases may lead them to re-explore previously chosen options, particularly in the later stages of the task. To capture these temporally varying exploratory behaviors from a new perspective, this study introduces a new evaluation metric called “Sequential Exploration Decay” (SED). This metric reflects the extent of information value decay by calculating the ratio of the number of consecutive four-different-deck events in the first 50 rounds (early exploration phase) to that in the last 50 rounds (late exploration phase).

By calculating the SED for the IGT data from 504 participants, the experimental data show that the actual SED ratio is 1.54923, whereas several existing decision-making models such as EV, PVLdelta, ORL, VPP, and VSE exhibit significantly higher SED ratios after simulation (EV and PVLdelta>5, ORL = 3.01339, VPP = 3.39871, VSE = 2.35052, all p-values < 0.05). This discrepancy suggests that although these models can simulate the decision-making process to some extent, they may fail to fully account for certain key psychological phenomena influencing human decision-making, such as forgetting and cognitive biases. Therefore, by introducing the SED metric, we aim to provide a more refined and accurate tool for analyzing and understanding the actual impact of these psychological phenomena on decision-making behaviors.

#### 3.2.2 Forgetting interval

Our analysis is conducted based on the hypothesis that negative feedback (e.g., suffering a large loss after choosing a particular deck) is typically perceived by participants as a high-information event, prompting them to avoid selecting that option again in the short term. However, as time progresses and new information is introduced (i.e., through interference-based forgetting; Ecker and Lewandowsky, 2012), the impact of this negative feedback may gradually diminish, leading participants to reselect the same deck. Therefore, we propose a new metric, the Forgetting Interval (FI), to quantify the extent to which participants forget information in the IGT. This metric measures the time (in terms of the number of trials) it takes for participants to reselect a deck after receiving negative feedback, providing a new dimension for computational models of the IGT to assess forgetting behaviors.

The four card decks (A, B, C, D) in the IGT are presented to participants with equal probability. Under the assumption of no preference or memory effects, the probability of randomly selecting any deck in each trial is 1/4. If participants have no specific preference or avoidance, the expected time interval before reselecting a deck after receiving negative feedback should be 4 trials, according to the expectation formula $\left(E=\overset{\infty }{\underset{n=1}{\sum }}{\left(\frac{1}{4}\right)}^{n}\cdot {\left(\frac{3}{4}\right)}^{n-1}\cdot n=4\right)$. However, by analyzing the data from 504 participants, we found that the average re-selection interval after receiving negative feedback is 6.24 trials (*SD* = 7.53), which is significantly higher than the expected random interval of 4 trials (*t*(503) = 6.69, *p* < 0.001). This finding indicates a notable delay in participants' decision-making behaviors when faced with unfavorable outcomes. In previous computational model designs, the phenomenon of reselecting an option after a period following a negative feedback event was often attributed to either exploitation or exploration strategies. However, such conventional explanations do not adequately account for why the FI is significantly shorter in older adults compared to younger adults. This observation suggests that models solely relying on exploitation and exploration strategies may overlook the critical role of memory and forgetting in decision-making processes, making it reasonable and necessary to introduce forgetting factors into computational models.

Our Forgetting Interval metric can thus be shown to capture the phenomenon of forgetting in the IGT and will serve as an important reference for subsequent model comparisons.

#### 3.2.3 Statistical model evaluation criteria: BIC, AIC, and free energy

To evaluate the goodness of fit and complexity of decision-making models, this study employs three statistical metrics: Bayesian Information Criterion (BIC), Akaike Information Criterion (AIC), and Free Energy (F).

The Bayesian Information Criterion (BIC), proposed by Schwarz (1978), aims to assess model performance. It is calculated using the sample size *n*, the number of model parameters *k*, and the maximum likelihood estimate $\hat{L}$. The formula is as follows:


$$\begin{array}{l} \text{BIC}=\ln\left(n\right)\cdot k-2\cdot \ln\left(\hat{L}\right) \end{array}$$ (8)

BIC imposes a larger penalty for model complexity, helping to prevent overfitting. A lower BIC value generally indicates a model that achieves a high goodness of fit while maintaining an appropriate level of complexity.

The Akaike Information Criterion (AIC), proposed by Akaike (1974), is calculated as:


$$\begin{array}{l} \text{AIC}=2k-2\ln\left(\hat{L}\right) \end{array}$$ (9)

AIC also evaluates the balance between goodness of fit and complexity, but compared to BIC, it imposes a smaller penalty for the number of parameters, making AIC more tolerant in model selection when dealing with models containing a large number of parameters, especially for large-sample data analysis.

Free Energy (F) is a metric used to assess the balance between fitting the data and model complexity, which is crucial for selecting the optimal model. Free Energy not only reflects the model's ability to fit the data but also considers its complexity, helping to avoid overfitting. In this study, we use Free Energy to comprehensively evaluate the performance of different models to ensure the selection of a model that accurately reflects the experimental data without being overly complex. The formula for calculating Free Energy is as follows:


$$\begin{array}{l} F=-0.5\times \text{SSE}-0.5\times ntot\log\left(2\pi \right)+0.5\times \log\det\left(Q\right)+S+dF \end{array}$$ (10)

Where SSE (sum of squared errors) measures the deviation between the model predictions and the actual data; *ntot* is the total number of parameters, reflecting the model's dimensionality and complexity together with the error term; logdet(*Q*) is the logarithm of the determinant of the prior precision matrix *Q*, representing the contribution of the prior distribution to the model complexity and the estimation of parameter uncertainty; *S* is the entropy of the model, describing the uncertainty in the posterior distribution of the parameters; and *dF* is the KL divergence between the posterior and prior distributions of the precision parameters (such as observation noise precision and state evolution noise precision), measuring the degree to which the model deviates from the prior assumptions.

This comprehensive evaluation method allows us to find the optimal balance between goodness of fit and model simplicity, thereby enabling more effective simulation and understanding of human decision-making behaviors.

#### 3.2.4 Approach for model and parameter recovery

Relying solely on traditional estimators such as the Akaike Information Criterion (AIC) or the Bayesian Information Criterion (BIC) may not be sufficient to comprehensively reflect a model's performance. This issue is particularly evident when inferring cognitive processes from model-estimated parameters. A common scenario is that a model with lower information loss or better data-fitting ability does not necessarily replicate actual choice behaviors more accurately, indicating that the model may still fail to capture the complexity of human cognition. Alternatively, similar behavioral patterns may arise from completely different parameter configurations, suggesting that the model's interpretability of human cognitive decision-making processes is limited. To address these challenges and ensure that the performance of our EEF model is at least comparable to, if not better than, that of previous models, we conduct analyses in Section 4.

First, for each model, we use each participant's actual decision data to set the best-fitting parameters of the model, and then employed these parameters to generate simulated IGT decision behaviors for the agent. Starting from initial prior knowledge, the agent received feedback for each round that followed the same rules as those of the participants, guided by the exploration and exploitation strategies as well as the consistency parameter. Next, we applied our EEF model and other competing models to the simulation-generated data, attempting to recover the initially set parameters from these data. By comparing the consistency between the parameters recovered from the simulated data and the original parameters, we evaluated the EEF model and previous models in terms of parameter recovery quality, stability, and reliability. Additionally, we compared the agent's simulated choices with the actual choices of participants. Through parameter recovery, we can identify the strengths and weaknesses of the model under specific decision contexts, thereby making targeted adjustments to the model parameters or algorithms to improve its performance and applicability in real-world settings.

## 4 Result

In this section, we explore the behavioral data of 504 participants in the Iowa Gambling Task, focusing specifically on the application effectiveness of the two newly introduced evaluation metrics—Sequential Exploration Decay (SED) and Forgetting Interval (FI)—in different decision-making models. By comparing the performance of the EEF model with that of five other models, we assess the impact of incorporating forgetting factors into model design on improving the accuracy of simulating real decision-making behaviors. Additionally, this section presents the models' performance in fitting actual choice data, providing empirical evidence to understand the effectiveness of each model in capturing the dynamics of human decision-making.

### 4.1 Forgetting phenomena—Sequential Exploration Decay and forgetting interval

In this subsection, we conduct a detailed comparison of the effects of the two newly proposed evaluation metrics—Sequential Exploration Decay (SED) and Forgetting Interval (FI)—in the EEF model and five other previously established models, using the IGT dataset from 504 participants as the basis.

The Sequential Exploration Decay (SED) metric introduced in this study is designed to measure the extent of information value decay and changes in exploratory behavior between the first 50 rounds and the last 50 rounds in the IGT.

**Figure 2:**
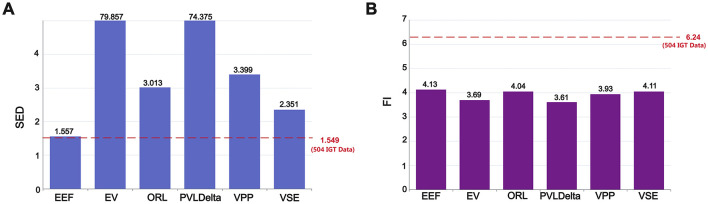
Model performance in forgetting phenomenon verification: **(A)** Performance in Sequential Exploration Decay (SED). **(B)** Performance in Forgetting Interval (FI). The red dashed line in **(A)** and **(B)** represents the SED and FI values derived from the IGT data of 504 participants. The EEF model is the closest model to these empirical values, indicating its higher accuracy in capturing human decision-making behavior.

Figure 2A presents the actual SED ratio based on data from 504 participants and the results simulated by different decision-making models. A ratio >1 signifies that exploration events are more frequent in the first half of the task than in the second half, thereby operationalising the gradual decay of information value. Conversely, a ratio close to 1 indicates that participants (or models) maintain a comparable level of exploration throughout. Hence, the proximity of the EEF bar to the empirical dashed line visually conveys its ability to reproduce the tempo of exploratory decline observed in humans.

The experimental data show that the actual SED ratio is 1.549, reflecting a gradual decrease in exploratory behavior throughout the task. As illustrated in the chart, there are significant differences in the SED ratios among different decision-making models: the SED ratios for the EV and PVLdelta models are much higher than the actual data, at 79.857 and 74.375, respectively. The SED ratios for the ORL, VPP, and VSE models are 3.013, 3.399, and 2.351, respectively—much lower than those of the EV and PVLdelta models but still significantly higher than the actual data. Statistical analysis using the chi-square test confirms that, except for the EEF model, the SED ratios of all other models differ significantly from the actual SED ratio(*p* < 0.001).

Such a stark contrast between early and late-stage exploration behaviors may indicate an excessive dependence and exploitation of early-acquired information in these models. This near-cessation of exploration in the later stages of the task contrasts with the gradually diminishing yet sustained exploration behavior observed in the actual data, highlighting a deficiency in the models' dynamic adaptability to simulate human decision-making. In contrast, the EEF model shows a more balanced pattern of exploration between the early and late stages, with an SED ratio of 1.557, which is very close to the actual data value of 1.549. This suggests that the EEF model, by incorporating forgetting, can better simulate the actual exploration dynamics and more accurately reflect human behavioral patterns and the phenomenon of forgetting in an ever-changing decision environment. It also indicates that the continued exploration observed in the later stages of the IGT may be due to some degree of forgetting of previously acquired information in human decision-making.

As for the Forgetting Interval (FI) showed in Figure 2B, a new evaluation metric designed to quantify participants' degree of forgetting after receiving negative feedback, an analysis of the data from 504 participants revealed that the actual FI is 6.24 trials, indicating that participants typically delay a period of time before reselecting the same deck after experiencing negative feedback.

In terms of model comparison, the EEF model's FI value is 4.13 trials. Although this differs from the actual data (6.24 trials), it is the closest to the real data among all models. Other models, such as the EV model (3.69 trials), the ORL model (4.04 trials), the PVL-Delta model (3.61 trials), the VPP model (3.93 trials), and the VSE model (4.11 trials), show FI values lower than the EEF model, indicating that their performance in simulating participants' forgetting behaviors deviates further from the actual situation. This result highlights the relative advantage of the EEF model in capturing decision behaviors related to forgetting. One-tailed Welch's *t*-tests further confirmed that the FI value of the EEF model was significantly higher than that of the EV, PVL-Delta, and VPP models (*p* < 0.01), indicating better alignment with the actual data. Although the differences between EEF and the ORL(*p* = 0.14) and VSE(*p* = 0.42) models were not statistically significant, the EEF model still achieved the closest approximation to the real data among all models. This observation suggests that further refining and adjusting the parameters of the forgetting mechanism in future model developments may improve model accuracy and practical applicability.

By comparing the performance of each model in terms of SED and FI, we found that the EEF model, which incorporates forgetting factors, achieves the closest alignment with the actual data across both metrics. This finding underscores the necessity of incorporating forgetting factors into models and highlights the EEF model's significant advantage in simulating the phenomenon of forgetting and its impact on human behavior. These results suggest that future research and model development should further explore and refine the mechanisms of forgetting to enhance the model's realism and predictive accuracy.

### 4.2 Statistical model evaluation (BIC, AIC, and free energy)

In this section, we conduct fixed-effects and random-effects analyses on the Bayesian Information Criterion (BIC), Akaike Information Criterion (AIC), and Free Energy (F) metrics for the EEF model and five other previously established models. The experiments are still based on the IGT dataset from 504 participants. It should be noted that using Free Energy (F) directly as a metric for model comparison may not allow for a direct comparison with other statistical metrics such as BIC and AIC, due to differences in their calculation methods and scales. To address this issue, this study apply a transformation to the Free Energy (F) metric by multiplying its value by –2, i.e., calculating −2 × *F* (Daunizeau et al., 2014). The purpose of this transformation is to align the dimensionality and value range of Free Energy (F) with those of BIC and AIC, thereby enabling a clearer and more rational comparison between models.

**Figure 3:**
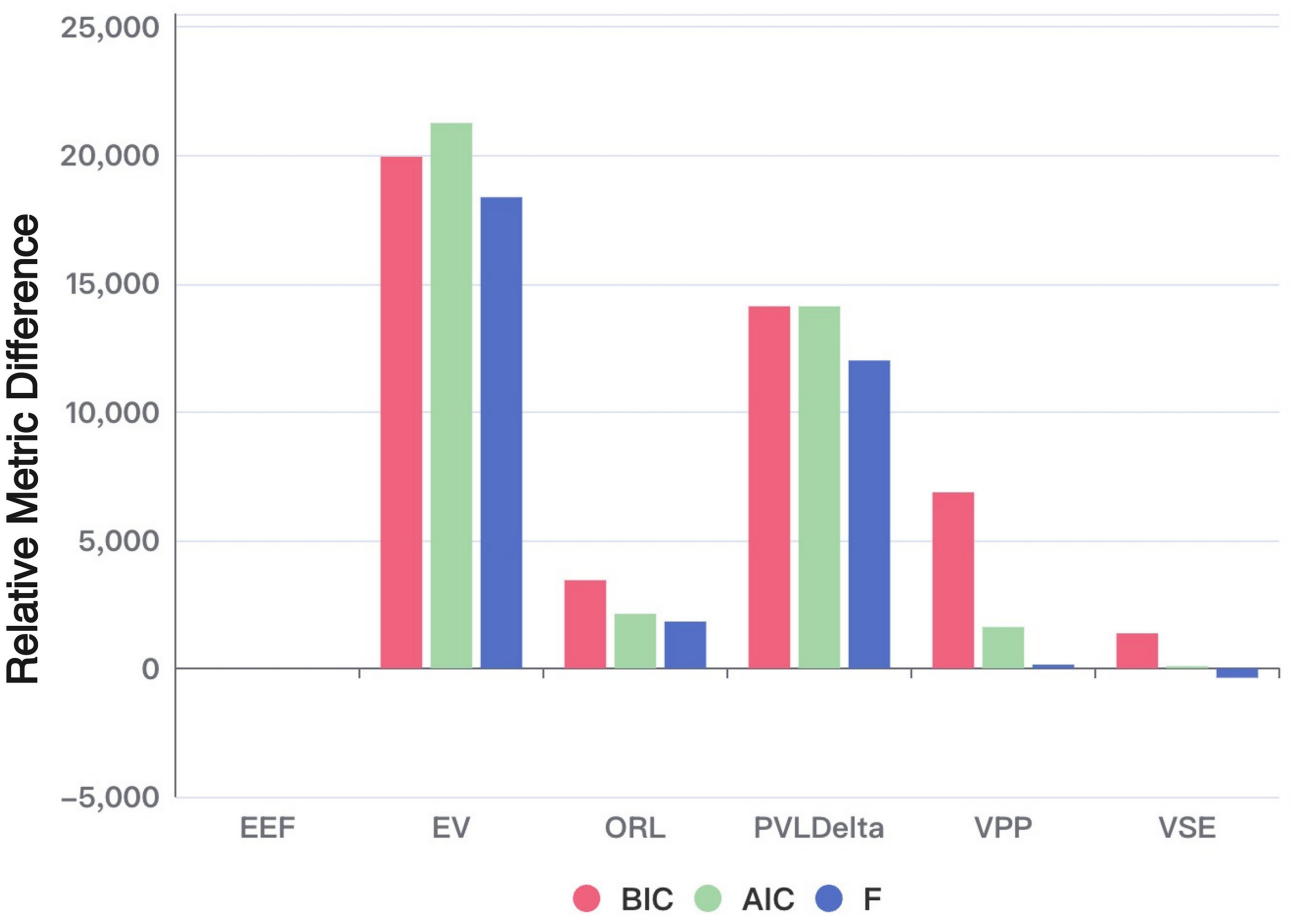
Performance of each model in terms of AIC, BIC, and free energy (relative to EEF). The bar chart illustrates the relative differences of each model compared to the baseline model (EEF). The values are computed using the formula: –2 times the sum of each metric (AIC, BIC, and Free Energy) subtracted by –2 times the sum of the corresponding metric for the baseline model EEF. Positive values indicate worse performance compared to EEF. The absolute values of each metric are also shown for reference.

Figure 3 uses the EEF model as the reference point and adopts a fixed-effects model comparison framework. In this context, positive bar values represent the amount of information loss incurred when selecting a model other than EEF. Because each bar reflects the difference between a given model's score and the EEF baseline, higher (positive) values indicate worse performance, while values at or below zero suggest comparable or superior performance.

As shown in the figure, the EEF model outperforms all other models across nearly all evaluation metrics. The only exception is the Free Energy criterion, where it performs slightly worse than the VSE model. Nonetheless, this marginal drawback is outweighed by its superior performance on the remaining metrics.

Under the Bayesian Model Selection framework, the EEF model achieved the highest estimated model frequency under both the BIC and AIC criteria, exceeding 70% and 40% respectively—well above the chance level (1/6) and other models. Notably, the confidence intervals of these estimates did not overlap with the chance level or other models, indicating a statistically robust group-level preference for the EEF model. These consistent results across multiple evaluation criteria provide strong Bayesian evidence for the robustness and generalizability of the EEF model in explaining human decision-making behavior in the IGT.

### 4.3 Simulation: model and parameter recovery

In this section, we present the results of the simulation experiments on model and parameter recovery. We focus on evaluating the EEF model's performance compared to the other five existing models in terms of prediction accuracy for fitting actual choice data and simulated choice data, as well as the quality of parameter recovery.

**Figure 4:**
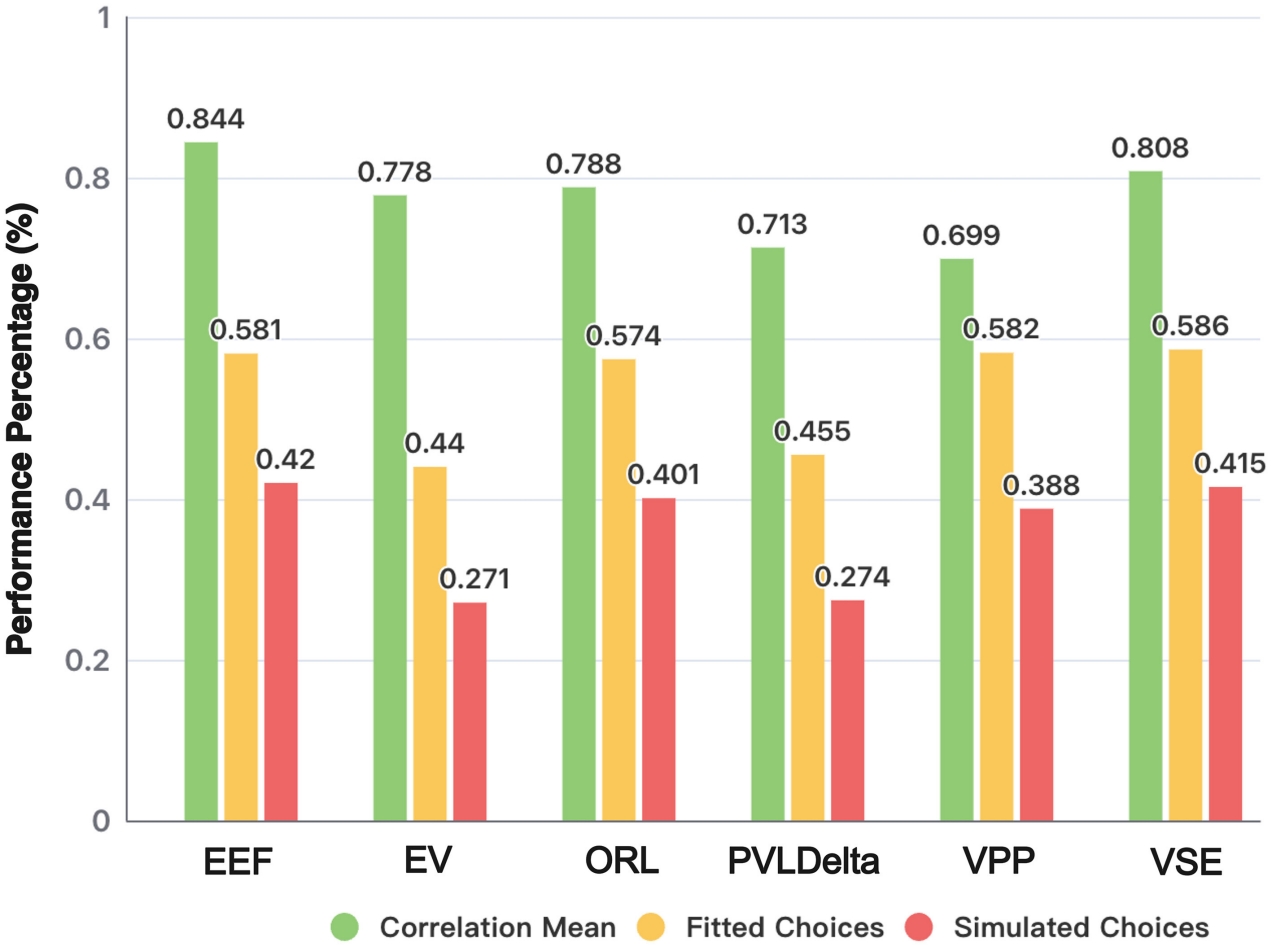
Model and parameter recovery acrossing models. Comparison of different models based on correlation mean, fitted choices, and simulated choices. Correlation mean evaluates the parameter recovery quality, while fitted and simulated choices indicate the models' ability to replicate participant behavior and generate consistent simulated choices.

Figure 4 shows the performance of three metrics that measure model recovery. In the analysis of fitting actual data (Fitted Choices), the EEF model achieved 58.13% accuracy, significantly outperforming the EV model (44.01%; *t* = 14.02, *p* < 0.0001) and the PVLΔ model (45.51%; *t* = 12.15, *p* < 0.0001). Its accuracy did not differ significantly from the ORL model (57.47%; *t* = 0.64, *p* = 0.521), nor from the VPP (58.21%; *t* = −0.08, *p* = 0.940), or VSE models (58.64%; *t* = −0.50, *p* = 0.618). Notably, the simplified EEF model achieves any equivalent fitting performance with fewer parameters than ORL, VPP, and VSE, underscoring its efficiency.

In the analysis comparing whether the choices derived from the agent (Simulated Choices) can replicate the original choice data, the EEF model achieved an accuracy of 41.977%—the highest among all six models—demonstrating strong performance. Two-tailed Welch's t-tests showed that its accuracy was significantly higher than that of EV [27.05%; *t*(73861) = 18.16, *p* < 0.0001], PVLDelta [27.43%; *t*(78709) = 17.28, *p* < 0.0001], and VPP [38.77%; *t*(100756) = 3.11, *p* = 0.0018], but did not differ significantly from ORL [40.14%; *t*(100632) = 1.80, *p* = 0.072] or VSE (41.55%; *t*(100773) = 0.50, *p* = 0.619). Despite using fewer free parameters, the EEF model still outperformed most alternatives, underscoring its robustness in replicating experimental behavior and its efficiency in simulating participants' decision-making processes. These findings confirm the effectiveness and stability of the EEF model in capturing complex cognitive dynamics.

**Figure 5:**
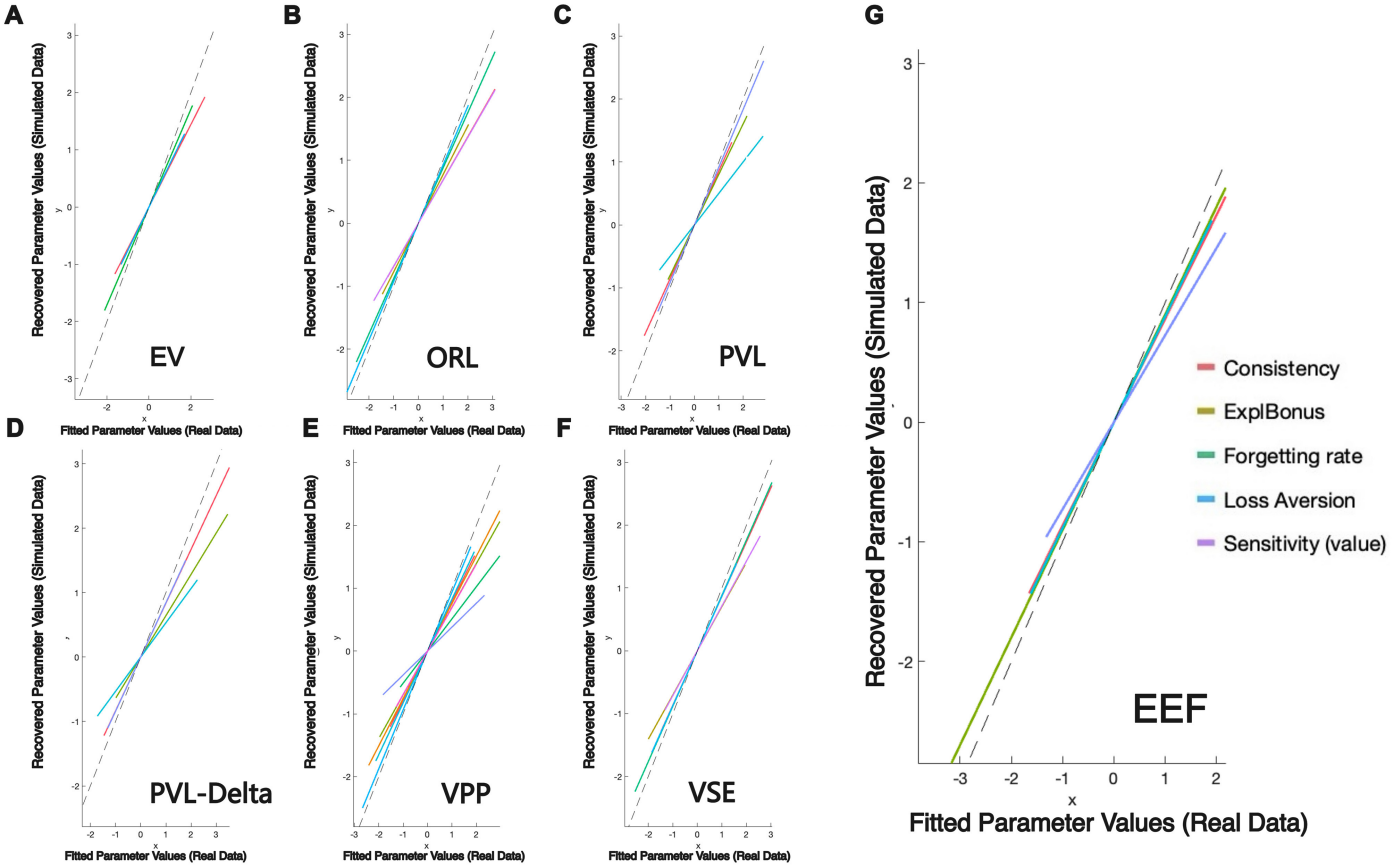
Parameter recovery analysis across. **(A)** EV Model, **(B)** ORL Model, **(C)** PVL Model, **(D)** PVL-Delta Model, **(E)** VPP Model, **(F)** VSE Model, and **(G)** EEF Model. The dashed line represents perfect equivalence between parameters fitted from the real data (x-axis) and recovered from simulated data (y-axis). EEF shows the highest consistency, with most parameters closely aligned along the ideal line, indicating superior recovery accuracy compared to other models.

Figure 5 presents the results of the parameter recovery analysis, providing a visual assessment of each model's ability to recover simulated parameters. All axes display *z*-scored parameter values, standardized by subtracting the sample mean and dividing by the standard deviation. This rescaling ensures that heterogeneous parameters are comparable and can be displayed on common axes.

As a result of this standardization, data points may assume negative values even when the original parameters (e.g., λ, θ∈[0, 1]) remain within their theoretical bounds. Negative *z*-scores simply indicate that a participant's parameter estimate falls below the sample mean.

The dashed line in each subplot marks perfect equivalence between parameters estimated from the real dataset (x-axis) and those recovered from simulated data (y-axis). Among all models, the EEF model shows the tightest clustering of points around this ideal line, demonstrating superior consistency and accuracy in parameter recovery.

Overall, the composite correlation coefficient for parameter recovery in the EEF model is 0.849 (range: 0.69–0.96), which is significantly higher than those of the other models. Specifically, the EV model yielded a composite coefficient of 0.787 (range: 0.75–0.86), the ORL model 0.791 (range: 0.67–0.87), and the VSE model 0.792 (range: 0.63–0.94). The PVLdelta and VPP models showed relatively inconsistent parameter recovery, with lower overall coefficients and broader variability across parameters (PVLdelta: 0.725, range: 0.52–0.86; VPP: 0.698, range: 0.38–0.94). Pairwise Fisher *z*-tests confirmed that the EEF model's recovery performance was significantly better than all other models (*p* values < 0.05 in all comparisons). Notably, the correlation coefficients for the *ForgettingRate* and *ExploreBonus* parameters in the EEF model reached 0.896 and 0.956, respectively, highlighting the model's strong explanatory and predictive power in capturing individual differences in decision-making behavior.

## 5 Discussion

### 5.1 Extension of EEF model with loss aversion

We further extended the EEF model by adding a loss aversion parameter (LA), ranging from 0 to 5, to the exploration module. This parameter reflects the tendency for individuals to form a more negative impression of an option after encountering negative feedback, thereby reducing its probability of being selected. We named this extended model the Exploitation and Exploration with Forgetting and Loss Aversion (EEFLA). Specifically, we made the following modification to the utility calculation in the exploitation module:


$$\begin{array}{l} V\left(t\right)=\text{Gain}{\left(t\right)}^{\theta }-\text{LA}*\text{Loss}{\left(t\right)}^{\theta } \end{array}$$ (11)

In addition, all other parts of the exploitation and exploration modules in the EEFLA model remain consistent with the original EEF model. We conducted a comprehensive comparative analysis between the EEF model and the EEFLA model.

**Figure 6:**
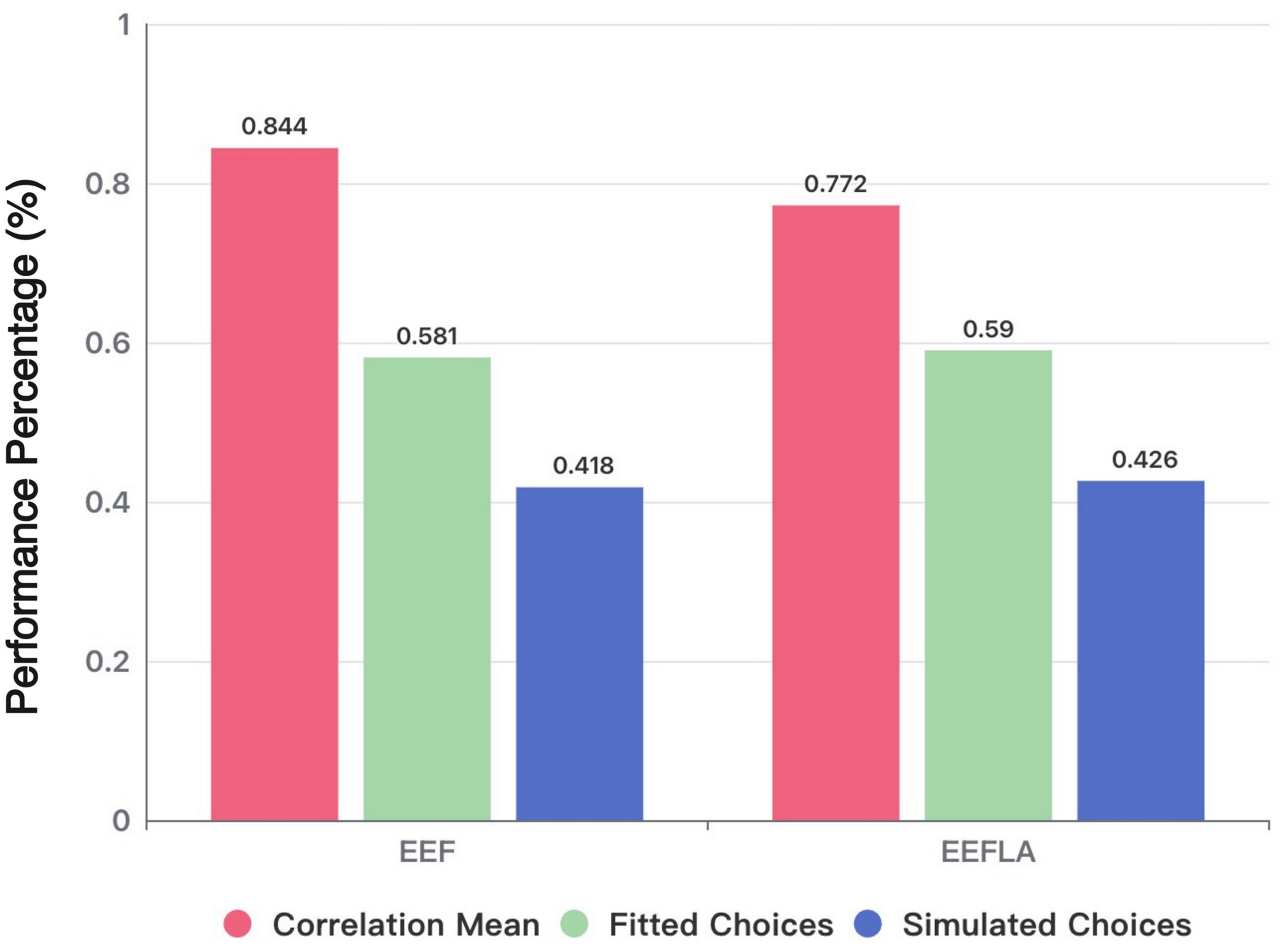
Model and parameter recovery across EEF and EEFLA model.

Figure 6 shows the performance of three metrics that measure model recovery across EEF and EEFLA. First, it is essential to highlight the EEFLA model's ability to fit actual data (Fitted Choices) and replicate original choices (Simulated Choices). As mentioned earlier, the EEF model outperformed previous models in terms of parameter recovery (Fitted Choices: 58.128%; Simulated Choices: 41.977%). After incorporating the loss aversion parameter, the EEFLA model showed even better performance on these two metrics, achieving a fit to actual data of 58.956% and replicating 42.613% of the original choices. This performance surpasses that of all models, including the EEF and the five comparison models. This result indicates that introducing the loss aversion parameter allows the model to better fit the original data.

**Figure 7:**
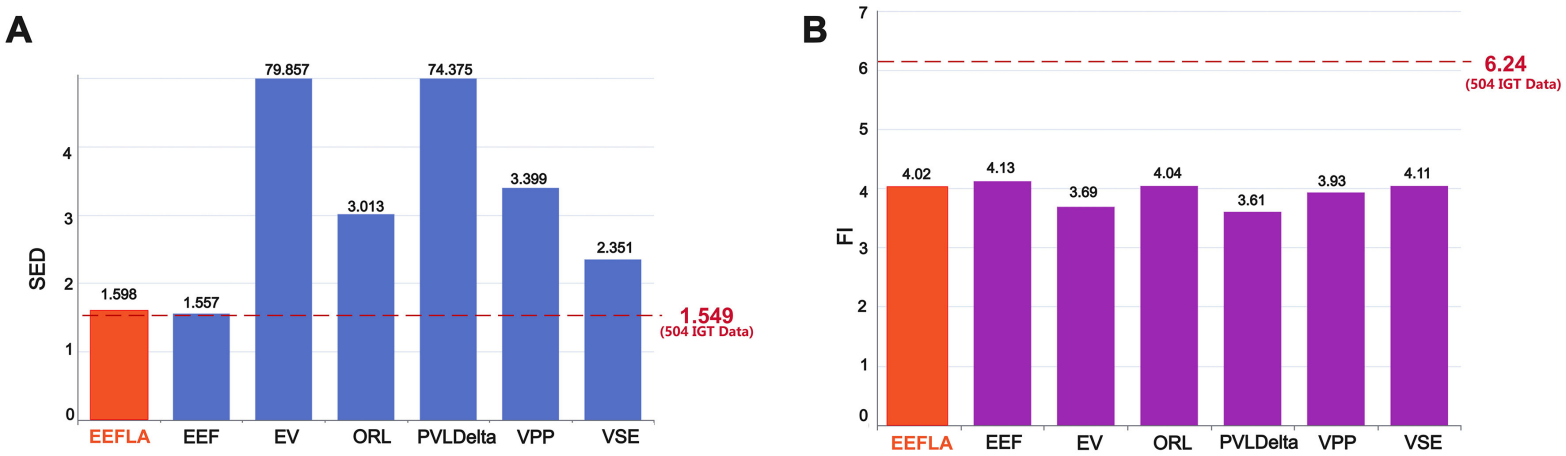
Model performance in forgetting phenomenon verification (EEFLA included): **(A)** Sequential Exploration Decay (SED). **(B)** Forgetting Interval (FI).

Figure 7 shows the comparison of SED and FI, and we can find that although the EEFLA model performed slightly worse than the EEF model (the SED of EEFLA is 1.598, with a difference of 0.049 from the original data, while the SED of EEF has a difference of only 0.008 from the original data; the FI of EEFLA is 4.02, with a difference of 2.22 from the original data, while the FI of EEF has a difference of 2.11), it still outperformed most of the other models. This indicates that the EEFLA model is still capable of effectively simulating human decision-making behaviors.

**Figure 8:**
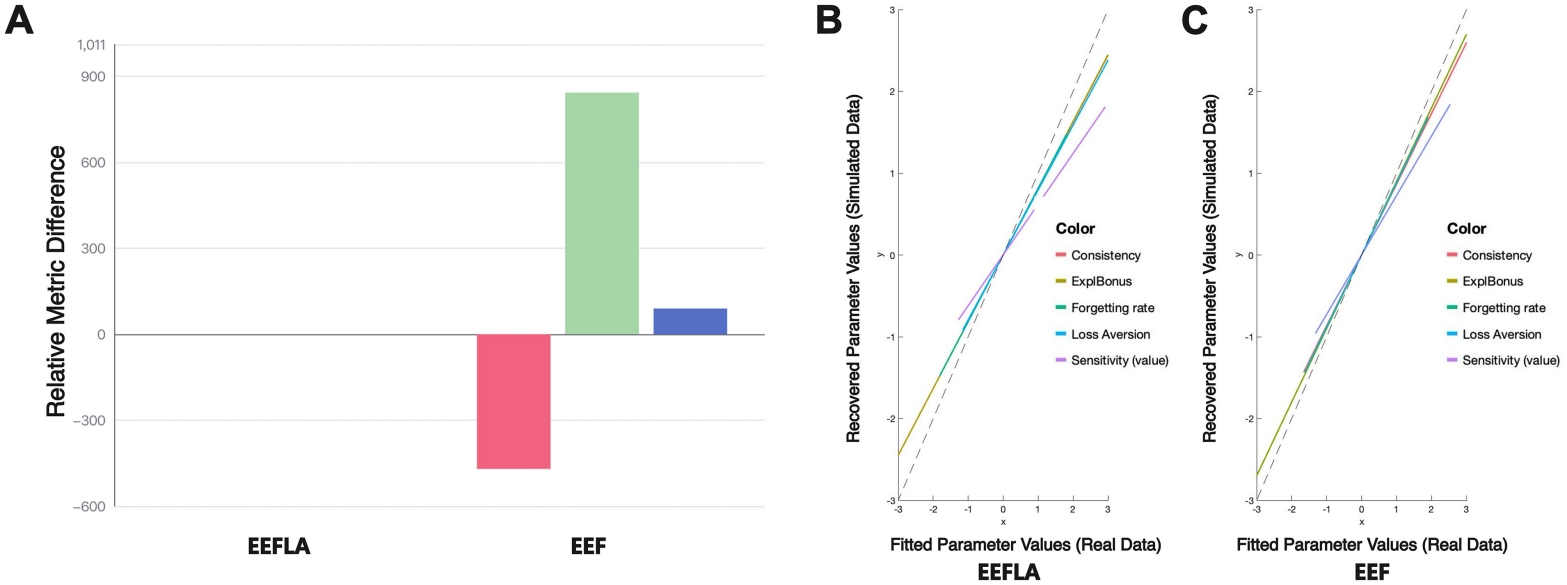
Performance comparison in EEF and EEFLA: **(A)** Performance in Terms of AIC, BIC, and Free Energy (Relative to EEFLA). **(B)** and **(C)** Performance in Parameter Recovery Across EEFLA and EEF.

However, it can be seen from Figure 8 that the performance of the EEFLA model is not outstanding in terms of parameter recovery and fixed-effects statistical model analysis. The composite correlation coefficient for EEFLA's parameter recovery is 0.785 (range: 0.58–0.93), which is lower than the EEF model's 0.849 (range: 0.69–0.96). Additionally, the EEFLA model did not perform well in the previously mentioned fixed-effects statistical model analysis.

In summary, although the performance of the EEFLA model with the loss aversion parameter is still inferior to that of the EEF model in many aspects, the EEFLA model may show superior performance when there is a higher demand for accurately reproducing human decision-making behaviors.

### 5.2 Capturing age- and gambling frequency-related IGT decision patterns with the EEF model

#### 5.2.1 Age-related differences in IGT performance

Isabel Gómez-Soria and colleagues found that older adults exhibit a higher forgetting rate when it comes to remembering the temporal order and task-related information (Gómez-Soria et al., 2021). This suggests that in tasks involving short-term memory, older adults experience faster information loss compared to younger individuals. However, in a study by Wood et al. (2005), there was no significant difference in IGT performance between older and younger groups, which might indicate that the two groups adopt different decision strategies. Therefore, we use the IGT dataset from Wood et al. (2005)'s study, which includes 90 younger adults (aged 18–40 years, average age = 23.04 years, *SD* = 5.88) and 63 older adults (aged 61–88 years, average age = 76.98 years, *SD* = 5.20), to evaluate the EEF model's effectiveness in capturing strategy differences among IGT participants and to investigate the forgetting patterns in older vs. younger adults.

**Figure 9:**
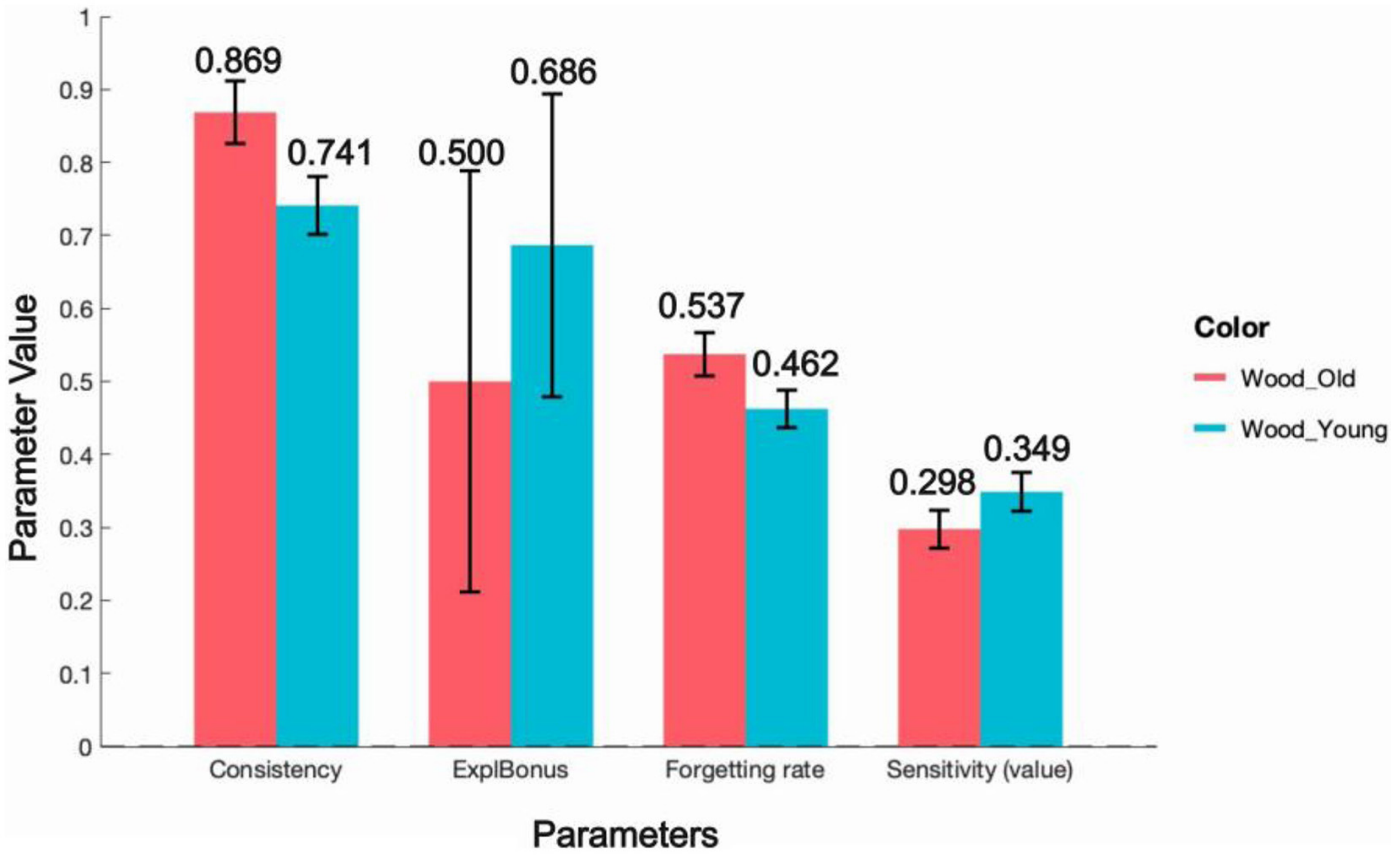
EEF-fitted parameters in young-old dataset.

When comparing the EEF model's fitted parameters for the IGT data of the older and younger groups, Figure 9 presents the average values of the model parameters along with error bars to display the range of these averages. We can see that the forgetting rate is higher in older adults compared to younger adults (Old: 0.537 ± 0.030; Young: 0.462 ± 0.026), which is consistent with the conclusions of Gómez-Soria et al. (2021).

At the same time, we also observe that older adults have significantly lower value sensitivity^1^ (Old: 0.298 ± 0.025; Young: 0.349 ± 0.026) and lower exploration reward (Old: 0.500 ± 0.289; Young: 0.686 ± 0.207) than younger adults, whereas the consistency parameter is significantly higher in older adults (Old: 0.869 ± 0.043; Young: 0.741 ± 0.039). This parameter pattern aligns well with Eppinger et al. (2012)'s findings that older adults show reduced striatal activity in the anticipation and processing of rewards, leading to decreased sensitivity to immediate rewards. It also corresponds with Sparrow and Spaniol (2016)'s observation that older adults are more inclined to choose familiar options rather than trying new or uncertain choices in tasks involving exploratory behaviors.

In summary, these fitted parameters reflect the different forgetting phenomena and distinct choice strategies between the older and younger groups in the IGT. This demonstrates that the EEF model can capture age-related variations in decision-making behaviors in both older and younger adult groups.

#### 5.2.2 Gambling frequency-related differences in IGT performance

We also apply the EEF model to a dataset from a study by Kildahl et al. (2020) that focused on decision-making behaviors among individuals with different gambling frequencies. To better investigate the characteristics exhibited by groups with varying gambling frequencies in the IGT using the EEF model, we merged the original four groups—less than once per month (38 participants), at least once per month (45 participants), once per week (29 participants), and multiple times per week (27 participants)—into two groups: low-frequency gamblers (Non-gamb: less than once per month and at least once per month, 83 participants) and high-frequency gamblers (Gamb: once per week and multiple times per week, 56 participants). The figure below shows a comparison of the EEF-fitted parameters for the 100-trial IGT results of the two groups.

**Figure 10:**
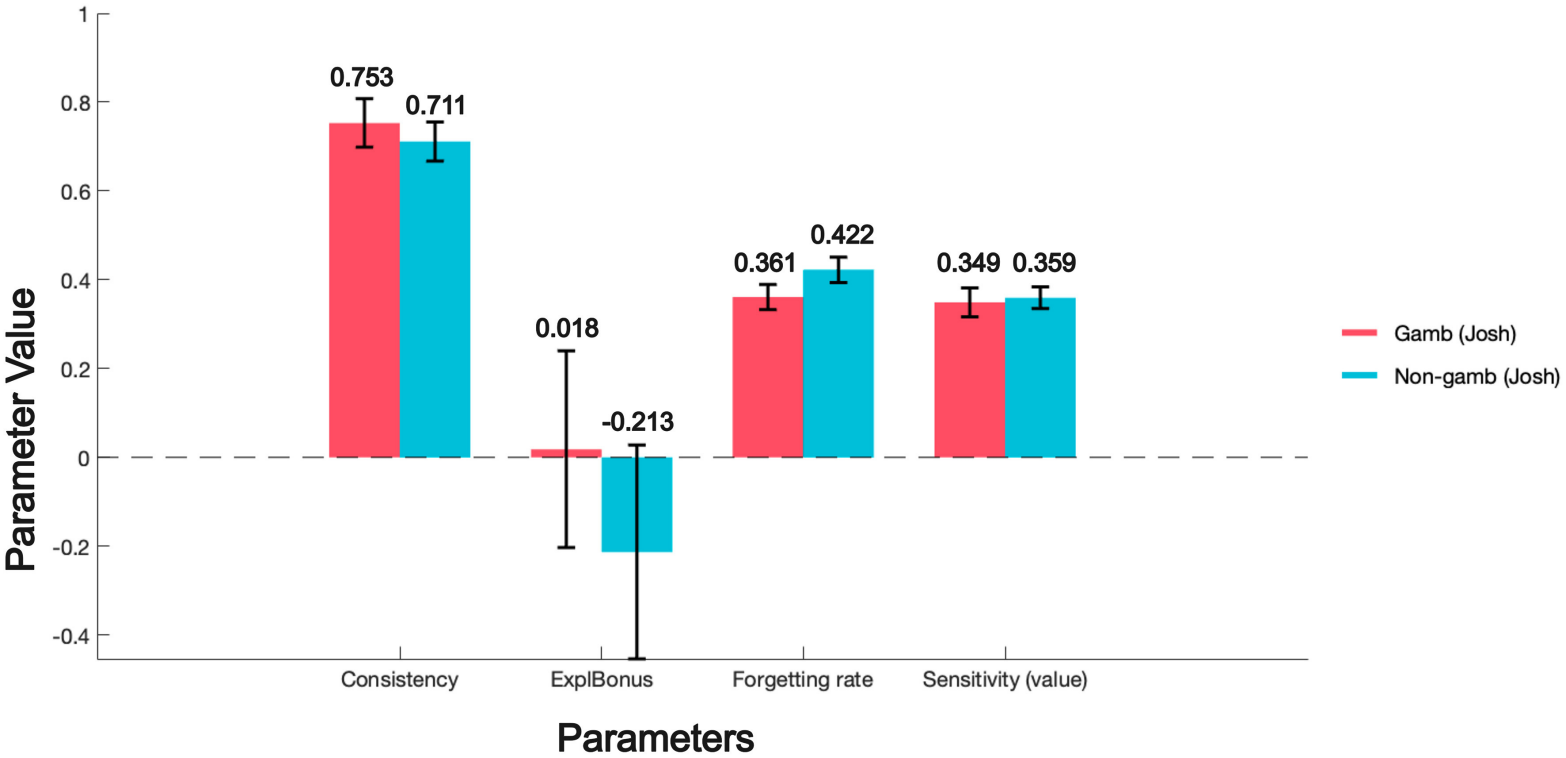
EEF-fitted parameters in gambling dataset.

We can observe from the Figure 10 that the Explore Bonus for low-frequency gamblers (Non-gamb) is negative (−0.213 ± 0.240), which contrasts sharply with that of high-frequency gamblers (0.018 ± 0.221). This indicates that individuals with low gambling frequency have a weaker tendency toward exploratory behavior compared to high-frequency gamblers, especially when exploration may yield uncertain outcomes; they may even avoid engaging in exploration. Such behavior suggests that low-frequency gamblers exhibit stronger risk aversion during exploration and prefer more stable choices over attempting new strategies.

Regarding the difference in Forgetting Rate, we can see that the forgetting rate of low-frequency gamblers is significantly higher than that of high-frequency gamblers (Gamb: 0.361 ± 0.028; Non-gamb: 0.422 ± 0.029). Although there is currently no specific research on the relationship between gambling frequency and short-term memory ability, some studies have explored the impact of games like Mahjong on short-term memory and working memory. A 12-week intervention study found that older adults with mild cognitive impairment showed significant improvements in executive functions, such as task-switching and multitasking abilities, after continuous participation in Mahjong activities (Zhang et al., 2020). Similarly, engaging in intellectual activities such as board games has been shown to reduce the risk of cognitive decline and improve memory and executive functions in older adults (Lee et al., 2018). In a related study, regular participation in board games was also found to enhance cognitive performance in terms of attention, memory, and problem-solving abilities among older adults (Yao, 2019). This can be analogized to the differences in forgetting rates fitted by the EEF model for different gambling populations. The complex rules and strategic operations required in certain types of gambling games may require participants to continuously process and memorize various types of information, thereby having a positive effect on short-term and working memory.

Although this conclusion is somewhat preliminary, we can still use the EEF model's analysis of IGT data to identify the behavioral characteristics of different populations in such decision-making tasks.

### 5.3 Conclusions and future research directions

This study proposes and validates a new computational model—the Exploitation and Exploration with Forgetting (EEF) model—which, for the first time, introduced a forgetting parameter in the context of the Iowa Gambling Task (IGT) and incorporated the initial conditions based on the first-choice data of 504 participants collected by Steingroever et al. (2015). By comparing the EEF model with previous models, we verified its effectiveness and addressed the gap in IGT research concerning the phenomenon of forgetting, revealing how individuals adjust their strategies in response to constantly changing environmental information.

Through the introduction and analysis of two new metrics, Sequential Exploration Decay (SED) and Forgetting Interval (FI), we found that the EEF model performs better than the other five models in capturing the phenomenon of forgetting in the IGT. The analysis of SED showed that the EEF model maintains a more balanced pattern of exploration behavior between the early and late stages of decision-making, and its SED value is closer to the real data compared to models like VSE and PVLdelta. This indicates that the EEF model more accurately simulates the process of information value decay over time, highlighting the central role of forgetting in exploratory behaviors. Furthermore, the EEF model's superior performance on the FI metric further demonstrates its effectiveness in simulating human behavior under the influence of negative feedback.

However, this study still has some limitations. Although the introduction of the forgetting parameter enhanced the model's explanatory power, its consistency with actual individual decision-making behavior needs further improvement in certain contexts. This may be due to the model's current inability to fully account for other complex influencing factors, such as emotional states and risk preferences. Modeling and integrating these factors present significant challenges, involving multi-dimensional data collection and complex algorithm design. In addition, although this study examined the behaviors of different age groups, a deeper exploration of specific populations (e.g., patients with mental disorders) regarding the differences in forgetting and decision-making behaviors requires more detailed research design and ethical approval. Regarding the neural mechanisms of forgetting, although existing research suggests that forgetting is closely related to the activities of the prefrontal cortex and hippocampus, integrating these neural mechanisms into our model remains a challenge. The acquisition and analysis of neurobiological data are complex and require interdisciplinary collaboration and advanced technical support.

Therefore, future research will focus on the following directions: First, refining the forgetting mechanism and exploring methods for dynamically adjusting the forgetting parameter to better simulate cognitive processes in various contexts. This may involve developing new algorithms or using machine learning techniques to automatically optimize model parameters. Second, applying the EEF model to more complex decision-making scenarios, such as multi-armed bandit tasks or real-life decision-making contexts, to verify its broader applicability and improve its generalizability. Finally, we plan to incorporate more psychological and neurobiological data, such as electroencephalography (EEG) and functional magnetic resonance imaging (fMRI), to further investigate the neural mechanisms of forgetting and decision behavior. By integrating these neural signals into the model, we hope to enhance the model's accuracy in simulating individual decision-making processes. Although this direction is highly complex, it has the potential to significantly improve the model's biological realism and predictive capability.

## Data Availability

Publicly available datasets were analyzed in this study. This data can be found here: https://osf.io/8t7rm/; https://osf.io/wxng6/.

## Footnotes

1 A notable point is that the significance threshold used here is 0.1.
