# Correction: Forgetting phenomena in the Iowa Gambling Task: a new computational model among diverse participants

**DOI:** 10.3389/fpsyg.2025.1642513

**Published:** 2025-07-03

**Authors:** Tiancheng Yang, Chenghan Xie, Xuehe Wang

**Affiliations:** ^1^School of Artificial Intelligence, Sun Yat-sen University, Zhuhai, China; ^2^College of Engineering, Department of Information Engineering, The Chinese University of Hong Kong, Hong Kong, China

**Keywords:** Forgetting Phenomena, Iowa Gambling Task, Exploitation and Exploration with Forgetting model, Sequential Exploration Decay, Forgetting Interval

In the published article, there was an error. Two sentences in section *5.1. Extension of EEF model with loss aversion, Paragraphs 4 and 5*, used outdated statistical values.

Corrections have been made to *5.1 Extension of EEF model with loss aversion, Paragraphs 4 and 5*. These paragraphs previously stated:

“Figure 7 shows the comparison of SED and FI, and we can find that although the EEFLA model performed slightly worse than the EEF model (the SED of EEFLA is 1.598, with a difference of 0.106 from the original data, while the SED of EEF has a difference of only 0.008 from the original data; the FI of EEFLA is 4.02, with a difference of 2.22 from the original data, while the FI of EEF has a difference of 2.07), it still outperformed the other models. This indicates that the EEFLA model is still capable of effectively simulating human decision-making behaviors.

However, it can be seen from Figure 8 that the performance of the EEFLA model is not outstanding in terms of parameter recovery and fixed-effects statistical model analysis. The average correlation coefficient for EEFLA's parameter recovery is 0.77155 (range: 0.60 – 0.93), which is lower than the EEF model's 0.8299 (range: 0.64 – 0.96). Additionally, the EEFLA model did not perform well in the previously mentioned fixed-effects statistical model analysis.”

The corrected paragraphs appear below:

“Figure 7 shows the comparison of SED and FI, and we can find that although the EEFLA model performed slightly worse than the EEF model (the SED of EEFLA is 1.598, with a difference of 0.049 from the original data, while the SED of EEF has a difference of only 0.008 from the original data; the FI of EEFLA is 4.02, with a difference of 2.22 from the original data, while the FI of EEF has a difference of 2.11), it still outperformed most of the other models. This indicates that the EEFLA model is still capable of effectively simulating human decision-making behaviors.

However, it can be seen from Figure 8 that the performance of the EEFLA model is not outstanding in terms of parameter recovery and fixed-effects statistical model analysis. The composite correlation coefficient for EEFLA's parameter recovery is 0.785 (range: 0.58–0.93), which is lower than the EEF model's 0.849 (range: 0.69–0.96). Additionally, the EEFLA model did not perform well in the previously mentioned fixed-effects statistical model analysis.”

The original article has been updated.

