# Correction: Forgetting phenomena in the Iowa Gambling Task: a new computational model among diverse participants

**DOI:** 10.3389/fpsyg.2026.1802516

**Published:** 2026-03-17

**Authors:** Tiancheng Yang, Chenghan Xie, Xuehe Wang

**Affiliations:** 1 School of Artificial Intelligence, Sun Yat-sen University, Zhuhai, China; 2 College of Engineering, Department of Information Engineering, The Chinese University of Hong Kong, Hong Kong, China

**Keywords:** forgetting phenomena, Iowa Gambling Task, exploitation and exploration with forgetting model, sequential exploration decay, forgetting interval

In the published article, the equation for the value of C in *Section 2.3 Consistency parameter and decision probability computation*, Paragraph 3 was erroneously given as *C* = 3β – 1.

The correct equation is *C* = 3^β^ – 1.

The original version of this article has been updated.

